# Natural Antioxidants: Fascinating or Mythical Biomolecules?

**DOI:** 10.3390/molecules15106905

**Published:** 2010-10-08

**Authors:** Ashwell R. Ndhlala, Mack Moyo, Johannes Van Staden

**Affiliations:** Research Centre for Plant Growth and Development, School of Biological and Conservation Sciences, University of KwaZulu-Natal Pietermaritzburg, Private Bag X01, Scottsville 3209, South Africa; E-Mails: ndhlala@ukzn.ac.za (A.R.N.); moyom@ukzn.ac.za (M.M.)

**Keywords:** antioxidants, bioavailability, bio-kinetics, free radicals, phenolic compounds, flavonoids, vitamins

## Abstract

Research on the use, properties, characteristics and sources of antioxidants especially phenolic compounds, flavonoids, vitamins, synthetic chemicals and some micronutrients began in the late 18^th^ century. Since then antioxidant research has received considerable attention and over a hundred thousand papers have been published on the subject. This has led to a rampant use of antioxidants in order to try to obtain and preserve optimal health. A number of nutraceuticals and food supplements are frequently fortified with synthetic or natural antioxidants. However, some research outcomes have led to the belief that antioxidants exist as mythical biomolecules. This review provides a critical evaluation of some common *in vitro* antioxidant capacity methods, and a discussion on the role and controversies surrounding non-enzymatic biomolecules, in particular phenolic compounds and non-phenolic compounds, in oxidative processes in an attempt of stemming the tidal wave that is threatening to swamp the concept of natural antioxidants.

## 1. Natural Antioxidants: an Overview

It is generally accepted that life evolved just after the Earth went through a violent raging bombardment that was accompanied by a high number of catastrophic events and the appearance of large quantities of molecular oxygen some 3.8 billion years ago, all of which favoured the formation of free radicals. Such harsh conditions could have easily have been the impetus for adaptation of early organisms as without the evolution of powerful antioxidants, life would never have survived. The origin of antioxidants date back to antiquity. The ancient Egyptians demonstrated a remarkable technical knowledge by preserving dead bodies with plants whose extracts are rich in phenolic compounds. The doors for research in oxidation/reduction reactions were opened by the notable oxidation of rubber during the late 1870s. By the 1940s, free radical autoxidation reactions were elucidated and several chain-breaking antioxidants were identified. By the late 1950s, it was shown that oxidation reactions are involved in aging and the progression of several diseases and it was proposed that antioxidant molecules may slow down the aging process, disease progression and prolong the lifespan [1]. This marked the first *in vivo* experiments involving feeding antioxidants to rodents and statistically significant effects of antioxidants increasing the lifespan were reported. This led to a battery of experiments to investigate sources, effects and toxicity of antioxidants thus producing a pool of more than 150,000 scientific papers on the subject to date.

The use of plants as food and medicinal remedies since ancient times is partially attributed to the biological efficacy of secondary metabolites that possess antioxidant activities such as phenolic compounds, vitamins C and E, and carotenoids. Phenolic compounds, which are derived from the shikimate and phenylpropanoid pathways, constitute a diverse and ubiquitous class of plant secondary metabolites characterised by an aromatic ring and one or more hydroxyl groups [2]. Middleton *et al*. [3] suggested that the biochemical and pharmacological activities of phenolic compounds, in particular flavonoids, are due to their long association with animal species and other organisms. Some of the proven biological activities include antimicrobial, antiviral, anti-inflammatory, antiallergic, vasodilatory effects and inhibition of lipid peroxidation [4]. 

Antioxidants are substances that, at low concentrations, prevent or retard the oxidation of easily oxidisable biomolecules such as lipids, proteins and DNA [5]. Ratnam *et al*. [6] simply defined antioxidants as substances which counteract free radicals, thus preventing oxidative damage. Two major groups of antioxidants are recognised, namely enzymatic and non-enzymatic antioxidants. Enzymatic antioxidants include the primary enzymes, superoxide dismutase, catalase and glutathione peroxidase, the secondary enzymes, glutathione reductase and glucose-6-phosphate dehydrogenase [6]. Non-enzymatic antioxidants, which are the focus of this review, are either water-soluble (vitamin C and phenolic compounds) or lipid-soluble (vitamin E and carotenoids) biomolecules [7]. Several other concepts are common in the field of antioxidant research including synergism, antagonism, oxidation retarders and bioavailability. Synergism relates to the mechanism in which a number of compounds (e.g. in plant extracts or foods), when present together in a system, have a more pronounced effect than would be derived from a simple additive concept [5]. Antagonism represents the opposite effect of synergism, where the combined effects of the compounds would be less pronounced compared to that derived from a simple additive effect. Antioxidant retarders are compounds that reduce the rate of oxidation without showing a distinct lag phase of the oxidation process [5]. Bioavailability relates to the absorption, distribution, metabolism and elimination of antioxidant compounds to produce a desired biological effect [8]. 

Phenolic compounds constitute the most abundant class of antioxidants with an estimated total dietary intake as high as 1 g/day, which is 10 times higher than the intake of vitamin C and 100 times that of vitamin E [9]. The antioxidant capacity of phenolic compounds has long been recognised for their strong chain-breaking actions and ability to scavenge radicals, thereby protecting cells against the detrimental effects of reactive oxygen species [3,7]. Reactive oxygen species (ROS) is a collective term for oxygen-centred radicals such as superoxide, hydroxyl and non-radical oxygen derivatives, namely hydrogen peroxide and singlet oxygen [10]. 

In humans the over-production of ROS can result in tissue injury and has been implicated in disease progression and oxidative damage of nucleic acids and proteins [3]. When there is a lack of antioxidants to quench the excess reactive free radicals, cardiovascular, cancer, neurodegenerative, Alzheimer’s and inflammatory diseases may develop in the body [11]. Due to the benefits of antioxidants, food and pharmaceutical products are normally supplemented with synthetic antioxidants such as butylated hydroxytoluene (BHT), butylated hydroxyanisole (BHA) and *tert*-butylhydroxyquinone (TBHQ). However, natural antioxidants from plant products may be more effective in reducing ROS levels compared to synthetic single dietary antioxidants due to the synergistic actions of a wide range of biomolecules such as vitamins C and E, phenolic compounds, carotenoids, terpenoids and phytomicronutrients [7,12,13]. In addition, the dietary intake of synthetic antioxidants could cause genotoxicity and carcinogenicity at high concentrations [1,14,15]. However, according to Becker *et al*. [5] and Shen *et al*. [16] the ascribed health effects of antioxidants in many studies are mainly based on *in vitro* assays which do not necessarily reflect the human physiological mechanisms *in vivo*. These controversies on the effectiveness of antioxidants have stimulated research on *in vivo* studies relating to their bioavailability to bridge the gap with *in vitro* observations. Available epidemiological research data tends to confirm the protective effects of phenolic compounds against cardiovascular diseases based on the use of reliable biomarkers [17,18]. On the other hand, the reported beneficial effects of phenolic compounds against cancers and neurodegenerative ailments such as Alzheimer’s disease are based largely on *in vitro* studies, due to lack of predictive biomarkers for such conditions [9]. The disparity between excellent *in vitro* successes and uncertain *in vivo* activities may be due to poor bioavailability coupled with low absorbability, and/or metabolism-derived loss-of-function of antioxidants [16]. The active compounds *in vivo* may be metabolites of native phenolic compounds found in plant products [9], thus creating a comparative mismatch to *in vitro* bioactivity. Another contributing factor could be that the effects observed from *in vitro* experimentation are based on much higher doses compared to those available in a normal human diet [9]. This review provides a critical evaluation of some common *in vitro* antioxidant capacity methods and a discussion on the role and controversies surrounding non-enzymatic biomolecules, in particular phenolic compounds and non-phenolic compounds, in oxidative processes. It is hoped that this review will counter the tidal wave that is threatening to diminish the concept and value of natural antioxidants. 

## 2. Protocols for Assaying Antioxidants

Various chemical *in vitro* assays have been developed to measure antioxidant capacities of plant products. A number of these assays depend on the generation of free radicals. Despite the recent popularity in antioxidant research, the lack of standardised assays to compare research results from different research groups has been a major challenge. As observed by Huang *et al*. [19], an examination of various antioxidant assays is required for the development of standard methods that are broadly applicable by researchers and industry. However, due to the complex nature of biological systems, there is no single universal method for measuring antioxidant capacity [20]. Based on reaction mechanisms involved, antioxidant capacity assays can be divided into two major groups; those based on hydrogen transfer (HAT) reactions and others involving single electron transfer (SET) reactions [19,20]. A third group consists of antioxidant capacity assays that involve both HAT and ET reaction mechanisms [20]. Assays that are based on HAT mechanisms measure competitive kinetics and are composed of a synthetic radical generator, oxidisable molecular probe and an antioxidant compound [19]. Since hydrogen atom transfer is a key step in the radical chain, HAT-based methods are more relevant to radical chain-breaking antioxidant capacity [19]. In contrast, SET-based assays involve one redox reaction in which the oxidant is also the probe for monitoring the reaction [19]. Single electron transfer-based assays involve two components in the reaction, *i.e.* the antioxidant and oxidant (also the probe) and follows the relationship [19]:

Probe (oxidant) + e (from antioxidant) → reduced probe + oxidised antioxidant

Colour change in the probe occurs when it removes an electron from the antioxidant, with the degree of colour change being proportional to the concentration of antioxidants in the reaction mixture. The reaction end-point is reached when the colour change stops [19]. 

Huang *et al*. [19] broadly classified antioxidant capacity assays based on reaction mechanisms as follows: SET [Folin-Ciocalteu (Folin-C), 1,1-diphenyl-2-picrylhydrazine (DPPH) radical scavenging, trolox equivalent antioxidant capacity (TEAC), ferric reducing ability of plasma (FRAP)]; and HAT [oxygen radical absorbance capacity (ORAC), *β*-carotene/linoleic acid model system and inhibition of phospholipid peroxidation]. Prior *et al.* [20] classified DPPH, TEAC and Folin-C antioxidant assays into a category that utilises both SET and HAT reaction mechanisms, though predominantly based on electron transfer. Some antioxidant assays have been developed to measure other reactive oxygen species e.g. the superoxide anion scavenging capacity assay. The following sections discuss some of the commonly used antioxidant capacity assays, the majority of which are done in our laboratory [21,22,23,24,25]. 

### 2.1. Electron transfer based assays

#### 2.1.1. Folin-Ciocalteu assay

The Folin-Ciocalteu method was initially developed by Folin and Denis [26] and Folin and Ciocalteu [27] for the determination of tyrosine (containing a phenol group) in proteins and modified by Singleton and Rossi Jr. [28] for the analysis of total phenolic compounds. The Folin-C assay in which the basic mechanism is a redox reaction, has been used for many years to quantify total soluble phenolic compounds in natural products using gallic acid as a standard. The reaction involves the reduction of phenols by a phosphomolybdic tungistic acid reagent. Phenolic compounds are oxidised in basic medium resulting in the formation of superioxide ion, which in turn reacts with molybdate to form molybdenum oxide (MoO^4+^) [27,28]. Molybdenum oxide has a very intensive absorbance at 725 nm. The total phenolic compounds are estimated by the reduction of the Folin-Ciocalteu phenol reagent. The reaction mixtures consisting of the sample, distilled water, 1 N Folin-Ciocalteu phenol reagent and 2-40% sodium carbonate are incubated at room temperature for 40 min. Absorbance is then measured at 725 nm. A reaction mixture containing the extracting solvent (usually 50% methanol) instead of samples is used as a blank. The concentration of phenolic compounds is calculated based on gallic acid equivalents (GAE). Phenolic compounds react with the Folin-C phenol reagent under alkaline conditions. The shortcoming for using the Folin-Ciocalteu method is that other non-phenolic compounds that are common in plant products, such as ascorbic acid, sugars, aromatic amines, organic acids and proteins are also reduced by the Folin-Ciocalteu phenol reagent [20,29]. Some inorganic compounds such as iron sulphate, potassium nitrite, sodium phosphate and manganese sulphate may also react with the phenol reagent resulting in overestimation of phenolic compound concentrations [20]. In order to obtain reliable results in the assay, the conditions specified by Singleton and Rossi [28] should be adhered to and gallic acid should be used as a reference standard [20]. Commercial tannic acid which used to be the common standard for the Folin-C method comprises a mixture of gallotannins from sumac (*Rhus semialata*), galls (Chinese gallotannins), aleppo oak (*Quercus infectoria*) and galls (Turkish gallotannins). The molecular weight of tannic acid was taken to be 1,294 g/mol, but has since been shown to be less than this value [30]. This is because of its heterogeneous mixture consisting of galloyl esters from Chinese galls and Turkish galls [31]. Tannic acid has since been replaced by gallic acid for use as a standard for phenolic assays.

#### 2.1.2. DPPH radical scavenging capacity

The 1,1-diphenyl-2-picrylhydrazyl (DPPH) assay for the determination of free radical scavenging capacity was first described by Bios [32] and subsequently modified by numerous researchers. The assay is one of the most extensively used to evaluate antioxidant activity of plant samples [11]. The DPPH free organic nitrogen radical is very stable, reacts with compounds that can donate hydrogen atoms and has a UV-vis absorption maximum at 515 nm [19]. The method is based on the scavenging of DPPH by antioxidants which upon a reduction reaction, decolourises the deep purple DPPH methanol solution. The chemical reaction mechanism of the DPPH assay is primarily based on SET reactions and hydrogen-atom abstraction is a marginal reaction [20]. The assay measures the reducing ability of antioxidants towards the DPPH radical using a UV-vis spectrophotometer. Alternatively, the antioxidant reducing ability can be evaluated by electron spin resonance. The greater the discolouration of the DPPH methanol solution, the lower the absorbance of the reaction mixture, thereby indicating significant free radical scavenging capacity. Sharma and Bhat [33] highlighted the wide concentration range of DPPH (22.5-250 µM), incubation time, reaction solvent and pH used by different laboratories as sources of variation in results. Based on research data, Sharma and Bhat [33] recommended the use of DPPH solution (50 µM) in methanol or buffered methanol depending on the solubility of the compound under investigation. A standard antioxidant, such as BHT is used as a positive control and a reaction mixture containing 50% aqueous methanol instead of sample solution is used as a negative control. The free radical scavenging activity as determined by the decolouration of the DPPH solution is calculated according to the formula:

% RSA = 100 × (1 – A_E_/A_D_)

where A_E_ is the absorbance of the reaction mixture containing the sample extract or standard antioxidant and A_D_ is the absorbance of the DPPH solution only.

The DPPH assay is commonly used because it is technically simple [19] and gives accurate and repeatable results [13]. The assay is valid to quantify samples with hydrophilic or lipophilic antioxidants. However, DPPH is a stable, long-lived nitrogen radical unlike radicals present in living organisms and has no similarity to the highly reactive and transient peroxyl radicals that are involved in lipid peroxidation [19]. Thus, antioxidants that react quickly with peroxyl radicals may react slowly or may be inert to the DPPH radical. Another limitation of the DPPH method is that some test compounds e.g. carotenoids, may have absorbance spectra that overlap with DPPH at 515 nm [13,20]. Steric accessibility is a major determinant of the reaction mechanisms, hence small molecules have higher apparent antioxidant capacity due to their better access to the DPPH radical site [20]. As explained by these authors, interpretation of results is difficult when small molecule reducing agents such as ascorbic acid are present in test extracts. This also applies to TEAC, in which the indicator radical (ABTS) is reduced either through hydrogen transfer or single electron transfer. 

#### 2.1.3. Trolox equivalent antioxidant capacity assay

The trolox equivalent antioxidant capacity (TEAC) assay, which was developed by Miller *et al*. [34] and adapted by Van der Berg *et al*. [35] and Re *et al*. [36] to involve the use of a pre-formed ABTS (2,2′-Azinobis-(3-ethylbenzothiazoline-6-sulfonic acid) radical, is widely used to measure total radical scavenging capacity. The assay is based on the discolouration of ABTS by antioxidant compounds, thus reflecting the amount of ABTS radicals that are scavenged within a fixed time period (6 min) in relation to that of 6-hydroxy-2,5,7,8-tetramethylchroman-2-carboxylic acid (Trolox). The oxidant is produced through a reaction between the ABTS radical (7 mM) and potassium persulfate (2.45 mM) in water. The reaction mixture which is allowed to stand at room temperature for 12-16 h before use, produces a dark blue solution. The resultant concentrated ABTS solution is diluted with ethanol or phosphate buffered saline (pH 7.4) to a final absorbance of 0.7 at 734 nm and 37 °C. At this absorbance, the concentration of the ABTS solution is approximately 47 µM. Ten µL of sample or trolox (a water-soluble analog of vitamin E used as the positive control) are added to 990 µL ABTS solution, and the reduction in absorbance is read at 734 nm after 1, 4 and 6 min. Total radical scavenging capacity of the sample is then calculated by relating the decrease in absorbance to that of trolox and is expressed in terms of the trolox-equivalent antioxidant capacity of the extract (TEAC/mg).

A limitation of this method is that the TEAC value characterises the capability of test extracts to react with the ABTS radical rather than to inhibit the oxidation process. The TEAC assay is highly dependent on the time of incubation and the ratio of sample quantity to ABTS radical concentration [37]. The ABTS radical is stable over a wide pH range and can be used to study pH effects on antioxidant mechanisms [38]. The ABTS radical is soluble in both aqueous and organic solvents, is not affected by ionic strength and can be used to measure the antioxidant capacity of hydrophilic and lipophilic compounds in test samples [39,40]. The radical, which has a low redox potential (0.68 V) is suitable for evaluating antioxidant capacity of phenolics due to their comparatively lower redox potentials. Many phenolic compounds can thus react with the ABTS radical because of this thermodynamic property [41]. 

#### 2.1.4. Ferric reducing ability of plasma

The ferric reducing ability of plasma (FRAP) assay was initially described by Benzie and Strain [42] for measuring reducing power in plasma and subsequently adapted and modified by numerous researchers to measure antioxidant power of botanical extracts in their ability to reduce Fe^3+^ to Fe^2+^. The assay is based on electron-transfer reactions, in which a ferric salt, potassium ferricyanide is used as an oxidant. The reaction mechanism involves the reduction of ferric 2,4,6-tripyridyl-*s*-triazine (TPTZ) to the coloured ferrous form. The redox potential of the Fe (III) salt is about 0.70 V and the assay is carried out under acidic (pH 3.6) conditions [19]. Sample extracts and standard antioxidants (e.g. ascorbic acid) are dissolved in 50% aqueous methanol to known concentrations. Potassium phosphate buffer (0.2 M, pH 7.2) and potassium ferricyanide (1% w/v) are then added to the sample solutions followed by incubation at 50 °C for 20 min. After the incubation period, trichloroacetic acid (10% w/v), distilled water and FeCl_3_ are added, followed by a second incubation at room temperature for 30 min in the dark. Absorbance is measured at 593 nm. A reaction mixture containing 50% aqueous methanol instead of the sample is used as the negative control. 

A major advantage of the FRAP assay is its simplicity, speed and robustness [20]. The FRAP assay is based on the hypothesis that the redox reaction proceeds rapidly and is complete within 4 min but this is not always true as some phenolic compounds react more slowly and require longer reaction times [20]. The assay is valid to quantify samples with hydrophilic or lipophilic antioxidants. One limitation of the FRAP assay is that any compound with a redox potential lower than 0.70 V may reduce iron, even though it may not behave as an antioxidant *in vivo* [13]. Protein and thiol antioxidants, such as glutathione cannot be measured by the FRAP assay. The FRAP assay is performed at a non-physiological pH [13], and the ferric ion is not relevant physiologically. 

### 2.2. Hydrogen transfer based assays

#### 2.2.1. Oxygen radical absorbance capacity 

The original oxygen radical absorbance capacity (ORAC) assay used the fluorescent *β*-phyco-erythrin (B-PE) as an oxidizable protein substrate (probe) and 2,2′-azobis(2-amidinopropane) dihydrochloride (AAPH) to generate peroxyl radicals [11]. However, B-PE is photobleached under fluorescence plate-reader conditions and reacts with phenolic compounds due to non-specific protein binding. Huang *et al.* [43] showed that upon exposure to excitation light at 491 nm, B-PE lost almost 53% of its fluorescence intensity over 35 min. To overcome the limitation of B-PE, the ORAC method was adapted to use a synthetic nonprotein probe, fluorescein (FL) [44,45], in a high-throughput assay using a multichannel liquid handling system coupled with a microplate fluorescence reader in 96-well format [43]. Added antioxidants compete with the substrate for the peroxyl radicals, thereby inhibiting or retarding FL oxidation. The assay, as recently described by Huang *et al*. [43], involves fluorescent measurements using a microplate fluorescence reader at a controlled temperature of 37 °C. The substrate (FL) decays in the presence of peroxyl radicals which are generated at a controlled rate by thermal decomposition of AAPH in an air-saturated solution. The fluorescence intensity is measured every min for 35 min under ambient conditions (pH 7.4, 37 °C) at the emission wavelength of 525 nm with extinction at 485 nm. Test samples (1.0 g/40 mL) are extracted in acetone/water (50:50, v/v) at room temperature on an orbital shaker for 1 h, followed by centrifugation at 8765 × *g* for 15 min. The supernatant is appropriately diluted to make different concentrations for the assay. The peroxyl radical generator, AAPH (153 mM) is dissolved in phosphate buffer (75 mM, pH 7.4) and kept on ice. Fluorescein stock solution (4.19 × 10^-3^ mM) is also prepared in phosphate buffer (75 mM, pH 7.4) and kept at 4 °C in the dark. Working fluorescein diluted solutions (8.16 × 10^−5^ mM) are prepared daily using the same phosphate buffer. Trolox stock solution (0.02 M) is prepared using phosphate buffer (75 mM, pH 7.4) and diluted with the same buffer to 50, 25, 12.5 and 6.25 µM working solutions for the standard curve. In the ORAC reaction, as fluorescein is consumed, its fluorescence intensity decreases. Fluorescein is photostable and exhibits no significant change in fluorescence intensity over 35 min. The assay uses the peroxyl radical which plays a key role in lipid oxidation in food and biological systems [19] and is the only method that combines inhibition time and degree of inhibition into one quantity using area-under-the-curve [46]. The ORAC assay also measures both hydrophilic and lipophilic chain-breaking antioxidant capacity. 

#### 2.2.2. *β*-carotene linoleic acid bleaching assay

The *β*-carotene linoleic acid bleaching assay as described by Miller [47] and modified by other researchers including Amarowicz *et al*. [48], measures the inhibition of the production of volatile organic compounds and the formation of conjugated diene hydroperoxidases due to linoleic acid oxidation which bleach the *β*-carotene in the emulsion. The reaction mechanism involves the bleaching of carotenoids via heat-induced oxidation and the resultant discolouration being inhibited or diminished by antioxidants that donate hydrogen atoms to quench radicals. The autoxidation of *β*-carotene is achieved by incubation in a water bath at 50 °C, making temperature control a critical parameter. Absorbance of *β*-carotene is measured at 470 nm. Sample extracts are dissolved in 50% aqueous methanol to a known concentration. *β*-Carotene (1.0 mg/mL) in chloroform is prepared in a brown bottle to prevent photo-oxidation. Excess chloroform is evaporated under vacuum leaving a thin film of *β*-carotene to which linoleic acid and Tween 20 are added. Oxygen-saturated distilled water is then added to the mixture to give a final *β*-carotene concentration of 20 µg/mL. The mixture is further saturated with oxygen by vigorous shaking to form an orange-coloured emulsion. The emulsion is transferred into test tubes containing a known concentration of the sample and standard antioxidant (positive control). Quercetin, BHT or ascorbic acid can be used as positive controls. A negative control consisting of 50% aqueous methanol in place of the sample, is used. A Tween 20 solution is used as a blank. A number of parameters can be calculated as indicators of inhibition kinetics. The rate of *β*-carotene bleaching is calculated using the following formula:

Rate of *β*-carotene bleaching = ln (*A_t=0_/A_t=t_*) ×1/t

where *A_t=0_* is the absorbance of the emulsion at 0 min; and *A_t=t_* is the absorbance at time *t*. The average rate of *β*-carotene bleaching is calculated based on rates at time *t*. The calculated average rates are used to determine the antioxidant activity (ANT) of the sample extracts and expressed as percentage inhibition of the rate of *β*-carotene bleaching using the formula:

% ANT = (*R*_control_ – *R*_sample_)/ *R*_control_ × 100

where *R*_control_ and *R*_sample_ represent the respective average *β*-carotene bleaching rates for the negative control and plant extracts. Antioxidant activity can be further expressed as the oxidation rate ratio (ORR) based on the equation:

ORR = *R*_sample_/ *R*_control_

Antioxidant activity (AA) is calculated as described by Braca *et al*. [49] and Moyo *et al*. [25] based on the inhibition of coupled oxidation of *β*-carotene and linoleic acid against the negative control at *t* = 60 min and *t* = 120 min using the formula:

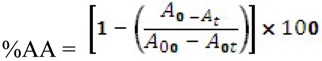

where *A_0_* is the absorbance of the sample extract at the beginning of incubation; *A_t_* is the absorbance at time *t* = 60 or 120 min for the sample extract; and *A_00_* and *A_0t_* represent the absorbance of the negative control (without sample extract) at the beginning of incubation and at time *t* = 60 or 120 min, respectively.

A major limitation of the assay is that the discolouration of *β*-carotene at 470 nm can occur through multiple pathways, thereby complicating the interpretation of results [20]. A substitute carotenoid, crocin, which bleaches only by the radical oxidation pathway, has been used in place of *β*-carotene [50,51,52]. However, a major drawback with the use of crocin is that it is not commercially available. Crocin must be extracted from saffron [50], hence the quality and purity of the compound may vary between laboratories. Extracted natural substrates usually contain endogenous chain-breaking antioxidants such as vitamin E, which can intervene in the testing procedure [37]. The use of commercially accessible individual compounds provides repeatable and reliable data between laboratories [37]. A major advantage of the carotenoid bleaching method is its applicability in both lipophilic and hydrophilic environments. Another advantage is that the carotenoid bleaching assay can detect either the antioxidant or pro-oxidant action of a compound under investigation [52]. Lastly, the carotenoid bleaching assay, using either *β*-carotene or crocin, can be adaptable to high-throughput technology such as the use of microplates [20].

#### 2.2.3. Inhibition of phospholipid peroxidation

Assays based on the inhibition of lipid peroxidation include model systems which involve oxidation substrates. The evaluation of antioxidants in model systems is based on measuring changes in the concentration of compounds being oxidized, on depletion of oxygen or on the formation of oxidation products [5,53]. Quantification of the loss of reactants, the formation of radicals and the formation of primary or secondary oxidation products, is the frequently used marker for lipid peroxidation depending on the stage of oxidation. Lipid oxidation involves concomitant formation and degradation of several products. Depletion of oxygen and electron spin resonance (ESR) detection of radicals, either directly or indirectly by spin trapping, can be used to follow the initial steps of oxidation. The assay uses brain dissected from 5-8 week-old Sprague Dawley rats (*Rattus norvegicus*). Rat brain (2 g) is homogenized in a chloroform:methanol mixture (2:1, v/v), followed by centrifugation at 402 × *g* for 5 min. The supernatant obtained is used as the source of phospholipids. The test run contains the phospholipids solution (50 μL), the sample extract (0.5 mL), methanol (50%, v/v) and FeSO_4_ (0.5 mL). The blank contains the phospholipid solution (50 μL) mixed with distilled water (0.5 mL) instead of the sample and methanol (0.2 mL, 50%). Ascorbic acid (0.5%) is used as the positive control. Incubation of the reaction mixture at 37 °C for 1 h is followed by the addition of thiobarbituric acid (TBA) (0.5 mL) and trichloroacetic acid (TCA) (4 mL). The solution is then heated in a boiling water bath for 15 min. After cooling the sample on ice, absorbance is read at 532 nm on a UV-vis spectrophotometer. A decrease in absorbance equates to low amounts of MDA, signifying higher protective ability against lipid peroxidation by antioxidants in the sample. 

Frankel [54] criticized the standard methods for the measurement of lipid oxidation because of peroxide value (POV) which is based on the formation of lipid hydroperoxides; the thiobarbituric acid reactive substances (TBARS) based on formation of secondary oxidation products; and the electrical conductivity caused by short-chained acids (Rancimat). Two different methods, an iodometric method and an iron/thiocynate method are the standards for POV, which often give different results. The TBARS test is often criticized for being unspecific because it measures the formation not only of malondialdehyde (MDA) but also of other oxo-compounds. Rancimat has the disadvantage of needing high temperatures which due to marked differences in energies of activation of different processes, may result in excess oxidation levels of the peroxides. For most applications, detection of oxidation products is a more sensitive method compared to the measurement of decrease in oxidation substrate concentration [5,55]. The relative protection against oxidation that is provided by various antioxidants depends on the type of substrate. In multiphase systems, the efficiency of antioxidants is greatly affected by solubility properties [5]. 

### 2.3. Other ROS scavenging capacity assays

#### 2.3.1. Superoxide anion scavenging activity assay

The superoxide anion scavenging capacity of samples is determined using the method outlined by Beauchamp and Fridovich [56]. Superoxide radicals are produced using a hypoxanthine-xanthine oxidase generating system coupled with nitroblue tetrazolium (NBT) reduction. The superoxide radicals are generated in a reaction mixture containing KH_2_PO_4_/KOH buffer (50 mM, pH 7.4), Na_2_EDTA solution in buffer (15 mM), hypoxanthine solution in buffer (3 mM), NBT solution in buffer (0.6 mM), xanthine oxidase in buffer (1 unit per 100 mL buffer) and sample (1.0 µg/25 µL buffer). Absorbance is measured at 540 nm 2.5 min after the addition of xanthine oxidase using a micro-plate reader. A reaction mixture containing buffer instead of sample is used as a blank. Superoxide scavenging activity is expressed as percent inhibition in comparison to the blank. A variation of the assay generates superoxide radicals using a phenazine methosulphate (PMS)-*β*-nicotinamide adenine dinucleotide reduced form, (NADH) system [57]. The reaction mixture for generating the superoxide radicals consists of Tris-HCl buffer (20 mM, pH 8.0) containing NBT (0.05 mM), PMS (0.01 mM, and the sample at varying concentrations. The reaction is started by the addition of NADH (0.078 mM) following an incubation period of 2 min. This method is, however, not suitable for quantifying nonenzymatic antioxidants.

### 2.4. Considerations for antioxidant capacity determinations

The hydrogen atom transfer mechanism is a key step in radical chain reactions, thus HAT-based methods are more relevant to the radical chain-breaking antioxidant capacity [19]. When assessing antioxidant capacity, it is important to use a variety of methods because of the complex heterogeneous nature of biological and food-related systems. Therefore, when selecting an antioxidant method, biological relevance of the reactants is a critical factor to consider [20]. As proposed by Becker *et al*. [5], evaluation of antioxidant capacity should be standardised on a four-step procedure as follows: (1) quantification and possible identification of phenolic compounds; (2) quantification of radical scavenging capacity, reduction potential, solvent effect; (3) evaluation of the ability to inhibit or halt lipid oxidation in model biological systems; and (4) storage studies using actual antioxidants incorporated in the food product, or human intervention studies using relevant biomarkers.

## 3. The Roles of Phenols as Antioxidants

The antioxidant properties of plant extracts have been attributed to their phenolic compound contents. A lot of work has been carried out since the early 1870s until now on the beneficial effects of phenolic compounds as natural antioxidants with well over 150,000 research papers associated with antioxidants available when searched through the Thomson Reuters Web of Knowledge (ISI Web of Knowledge), Medical Literature Analysis and Retrieval System Online (MEDLINE) and SciFinder [16]. Some of the research has lead to the identification and/or isolation of potent simple and polymeric phenolic compounds [58,59,60,61,62,63,64]. Through epidemiological studies, phenolic compounds have been shown to act as natural antioxidants by helping to neutralize free radicals and as metal chelating agents [25,65,66,67,68,69,70,71,72,73]. Phenolic compounds such as gallic acid, trans-resveratrol, fisetin, quercetin and its glycoside rutin have been reported to have strong antioxidant activity in the fluorimetric assay [74,75].

**Figure 1:**
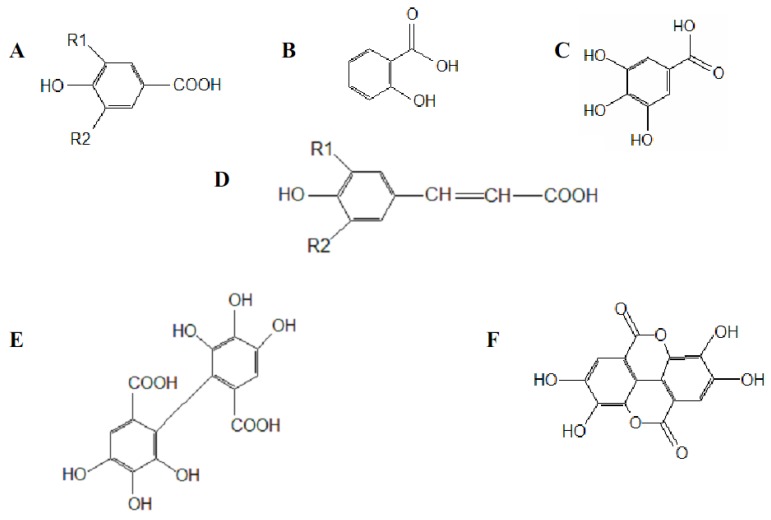
(A) *p*-Hydroxybenzoic; R1= R2=H, vanillic; R1= methoxy group, R2=H, protocatechuic acid; R1= OH, R2=H and syringic acid; R1= R2 = methoxy group. (B) Salicylic acid. (C) Gallic acid. (D) *p*-Coumaric acid; R1= R2 = H, caffeic acid; R1= OH, R2 = H, ferulic acid; R1= methoxy group, R2 = H and sinapic acid; R1= R2 = methoxy group. (E) Hexahydroxydiphenic acid. (F) Ellagic acid.

The multiple hydroxyl groups in the chemical structure of polyphenols make them ideal for free radical-scavenging reactions and as metal chelating agents [75]. The arrangement of the hydroxyl groups around the phenolic molecule is also important for antioxidant reactions [76]. Figure 1 presents some simple and dimeric phenolic compounds whose structures confer high antioxidant activity. Studies aimed at understanding the structure-activity relationships have been used as a theoretical method for predicting antioxidant activity [76]. 

**Figure 2:**
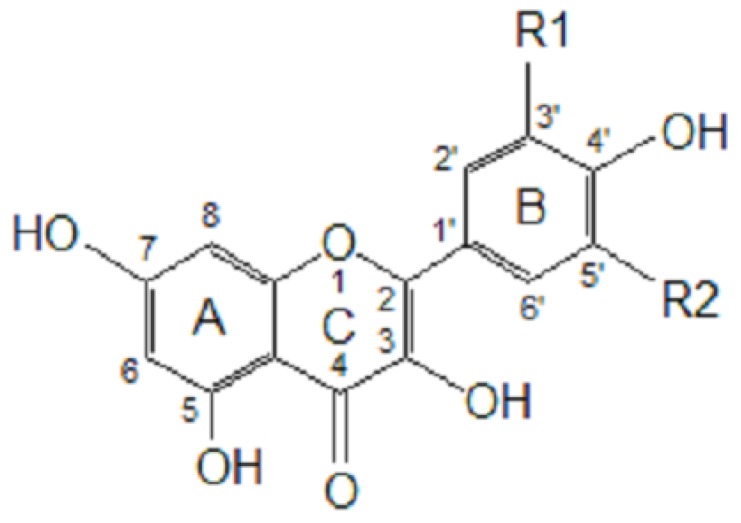
Flavonoid skeleton showing rings A, B and C and the numbering. Kaempferol, R1= H, R2=H; quercetin, R1= OH, R2= H; myricetin, R1=OH, R2= OH.

As reported by Hagerman *et al*. [77], the antioxidant potential of phenolic acids improves as the number of hydroxyl and methoxyl groups increases, hence the higher antioxidant ability of condensed and hydrolyzable tannins at quenching peroxyl radicals over simple phenols. Phenolic compounds comprise of a large diversity of compounds among which flavonoids and several classes of non-flavonoids are abundant. The numbering system for the flavonoid skeleton is shown in Figure 2. Flavonoids are a diverse group of secondary plant metabolites that are characterized by the presence of a C15-(C6-C3-C6) flavone nucleus that is based on a heterocyclic ring system derived from phenylalanine (ring B) and polyketide biosynthesis (ring A) linked through an oxygen containing pyran or pyrone ring (ring C) [78,79]. Flavonoids are common as oligomers and polymers (condensed tannin or proanthocyanidins) and are divided into several classes according to the degree of oxidation of the oxygen heterocycle into flavones, flavonols, isoflavones, anthocyanidins, flavanols and flavanones [29]. Stronger superoxide-scavenging activity in polymeric flavonoids with higher degrees of polymerisation has been reported [29]. 

## 4. Other (Endogenous) Types of Antioxidants

Besides exogenic phenolic compounds, biological systems have evolved several antioxidant mechanisms to control oxidative processes. These systems include antioxidant enzymes such as superoxide dismutases (SOD), catalases and peroxidases which are involved in the catalyzed removal of superoxide and hydrogen peroxide [5]. In addition to the three enzymes listed above, glutathione transferase, ceruloplasmin, hemoxygenase and possibly several other enzymes, may participate in enzymatic control of oxygen radicals and their products [80]. 

Apart from enzymatic antioxidant systems, non-enzymatic systems also exist within biological systems. These include vitamins E and C and glutathione. Vitamin E (tocopherols and tocotrienols) is the major lipid-soluble chain-breaking antioxidant in body tissues which plays an important role in the first line of defence of membranes against oxidative damage at early stages of free radical attack. The primary activity of vitamin E is to trap peroxy radicals in cellular membranes. This has led to the credence given to vitamin E in preventing or minimizing free radical damage associated with cancer, cardiovascular disease, premature aging, cataracts, and strenuous exercise [81].

Vitamin C (ascorbic acid) is a water-soluble antioxidant that is involved in the reduction of radicals from a variety of sources. Vitamin C participates in recycling radicals produced by oxidation of vitamin E. Vitamin C functions as a pro-oxidant under certain circumstances and sometimes produces oxygen as by-products that can cause damage to cells [82].

Glutathione is one of the most important intracellular barricades against damage by reactive oxygen species. The cysteine on the glutathione molecule provides an exposed free sulphydryl group that is very reactive, providing an abundant target for radical attack. Reaction with radicals oxidizes glutathione but the reduced form is regenerated in a redox cycle that involves glutathione reductase and the electron acceptor NADPH [80]. 

**Figure 3:**
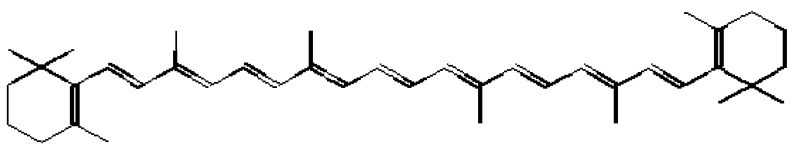
The chemical structure of *β*–carotene, one of the most abundant carotenoids.

In addition to vitamin E, vitamin C and glutathione, several other micromolecules such as bilirubin, uric acid, carotenoids and numerous other small molecules can function as antioxidants [82,83]. Carotenoids are naturally occurring lipophilic compounds with *β*-carotene (Figure 3) being the most abundant. They are characterised by an extended system of conjugated double bonds which are responsible for their antioxidant properties. Carotenoids protect biological systems against singlet oxygen mediated damage by direct quenching. They are also capable of inhibiting free radical reactions [84]. Some basic amino acids such as lysine and arginine and acidic amino acids such as aspartate and glutamate, have been reported to exhibit antioxidant activity by chelating metal ions [85,86]. It was reported that the basic amino acid, histidine, may behave both as a radical-scavenger and a metal-chelator due to its imidazole ring [86]. The sequence of amino acids in a protein molecule is the major factor that determines the antioxidative properties of proteins [85,86].

## 5. Antioxidants in Foods, Beverages and Drug Products

Herbs, fruits, vegetables, spices and other plant materials rich in phenolic compounds are of growing interest in the food industry because they retard oxidative degradation of lipids and thereby improve the quality and nutritional value of food [87]. Both synthetic and natural phenolic compounds have recently received a lot of attention as food additives [59,88,89,90,91,92]. Some commonly used antioxidants include BHA, BHT, TBHQ, tocopherol and caffeic acid. The use of these antioxidants as food additives, however, is limited for various reasons which include the polarity and size of the molecules [59,3]. 

Red wine has been regarded as beneficiary to humans, probably because of the protective effects on health compared to other alcoholic beverages [94,95]. For example, in the “French paradox”, there is a general consensus which gives credit to the wide consumption of red wine for the lower prevalence of coronary heart disease amongst the French population, despite having a diet relatively rich in saturated fats [96]. This is possibly due to the phenolic compounds in red wine that help prevent oxidative stress-related diseases such as cancer [97,98]. Alén-Ruiz *et al*. [99] reported the presence of various anthocyanins, hydroxycinnamic acids and stilbenes in different wine varieties and suggested that not all phenolic compounds possess the same biological activity. Thus the composition of red wines can be strongly affected both quantitatively and qualitatively by the particular grape cultivar, grape ripeness, environmental factors and technological procedures in wine-making. Wine is a complex matrix which contains several volatile compounds as well as some micronutrients such as protein, amino acids and sugars, thus making each wine different from the others [100]. 

Besides wine, fruit juices and milk are beverages that offer a mixture of several antioxidant compounds such as vitamin C, carotenoids and phenolic compounds, together with proteins and calcium (from the milk). Fruits provide the largest contributions of antioxidants in the diet, mainly because of the abundance of vitamins, phenolic compounds and carotenoids. Other studies have shown that some fractions of milk (whey, caseins, lactoferrin and albumin) have antioxidant activities [101].

The most abundant phenolic compounds in foods are caffeic and ferulic acids (in Figure 1 and Figure 2) [29]. Ferulic acid is mainly obtained from dietary fiber, sources of which include wheat bran. Caffeic acid occurs mainly as esters (chlorogenic acid) and is largely obtained from coffee, fruits and vegetables [63,102,103,104]. A cup of coffee (200 mL) contains 50-150 mg of caffeic acid esters [29]. Blackberry, raspberry, strawberry and brandy (aged in oak barrels) contain ellagitannins, gallotannins and simple gallic acid [105,106,107]. Flavonoids have been reported to be the major phenolic compounds in onions [108]. However, the health benefits and nutritional effects of food, fruit produces and beverages depend on the type of processing methods used during manufacturing, packaging, post harvest handling and storage [109].

In drug preparations, the potency of antioxidants is determined by many factors such as the chemical reactivity of the antioxidant molecule towards radicals, localization of antioxidants, concentration and mobility at the micro-environment, fate of antioxidant-derived radicals, interaction with other antioxidants, absorption, distribution, retention, metabolism and safety [110]. The potency of a novel antioxidant molecule, 2,3-dihydro-5-hydroxy-2,2-dipentyl-4,6-di-*tert*-butylbenzofuran (BO-653), was reported to surpass the activities of α-tocopherol and probucol in suppressing the oxidative modification of LDL [110]. 

For a phenolic compound or other molecule to be suitable for use as an antioxidant drug or food additive, it should have high reactivity towards the lipid peroxyl radical, a chain carrying species in lipid peroxidation [110]. In other words, it should react with the peroxyl radical much faster than the lipids which are present at much higher concentrations than the antioxidant itself. There is an increasing number of reports suggesting a non-classical role of antioxidants, beyond the antioxidant functions, that are not explained by redox reactions alone but by other mechanisms e.g. vitamin E also exerts a broad range of effects that can promote vascular homeostasis [111]. Another classical example involves the interaction of soy isoflavones with estrogen receptors and the effects of these compounds on the endocrine function. These interactions best explain the prevention by isoflavones of bone resorption in postmenopausal women without direct redox reactions [9]. 

Besides the health benefits, antioxidants are required for preserving food for prolonged storage, transport and prolonged shelf-life in supermarkets. This has resulted in the global increase in the consumption of numerous vitamins, trace elements and micronutrients with well defined biochemical functions (such as ascorbate, BHT and BHA). Ascorbic acid, BHT and BHA have already been re-branded as ‘universal antioxidants’ and are available in pharmaceutical formulations as supplements [1]. According to Gutteridge and Halliwell [1] foods naturally containing antioxidants but not super-rich in calories, namely fruits, vegetables and grains, help maintain human health and delay disease onset. It should be noted that several factors including pre-treatments and age of food samples could affect their antioxidant capacity [112].

## 6. When Do Antioxidants Act as Toxins?

Many researchers in the field of antioxidants focus only on the beneficiary effects of antioxidants and only a few have done research on their toxicity. This is mainly because there is a general belief that natural products especially of plant origin are safe. Moreover, most diseases are associated directly or indirectly with oxidative stress and use of antioxidant-rich foods or antioxidant food supplements are fast becoming popular. This could result in abuse, and/or careless application of antioxidant drugs, foods and food supplements. Toxicologists have recently warned against the delusive safety of natural products, in particular antioxidants of plant origin [113]. The side effects or risks of toxicity of antioxidants should not be taken lightly. 

To ascertain the safety of antioxidants, several questions have to be answered. These include; what are the effects of overconsumption of antioxidant and/or supplements with regards to their interactions with other micronutrients; are they bio-available; if so, how efficient is absorption of antioxidants in the gastrointestinal tract; what is the fate of the antioxidant and/or its metabolites; what are the effects of stomachic acids on the chemical structures and or bioactivity of antioxidants? As it stands, there are a lot of uncertainties as to the negative effects of antioxidants. Urso and Clarkson [114] reported that antioxidant supplements could have a negative effect on recovery from muscle damaging exercise. Their findings were, however, in contradiction to the study by Jakeman and Maxwell [115] that used a similar exercise stress and found that vitamin C supplementation prior to the exercise resulted in a faster recovery of muscle strength.

High levels of vitamin E have been reported to exacerbate impaired blood coagulation [116]. Van Haaften *et al*. [117], reported that vitamin E and several of its esters inhibit glutathione S-transferase P 1-1 (GST P 1-1). More so, pro-oxidant effects of vitamins C and E have also been reported [113]. The pro-oxidant activity is caused by the reducing powers of these two vitamins on some transition state metals. This reduction facilitates the priming of free radical chain reactions. These pro-oxidant effects are reported to be involved in fatal myocardial infarctions that were observed in a clinical study with vitamin E supplements [113,118].

Bast and Haenen [113] reported some toxic metabolites of vitamin E. They discovered quinones, which are metabolites of vitamin E metabolism. The quinones are generated when vitamin E exerts its antioxidant activity and are highly cytotoxic due to their ability to generate oxygen radicals, oxidise cellular components and form adducts with cellular thiols [113]. Another metabolite of vitamin E, 2,7,8-trimethyl-2-(*β*-carboxyethyl)-6-hydroxychroman, has been reported to possess a strong nutraceutical effect [113,119]. *γ*-Tocopherol, another form of vitamin E is an effective inhibitor of the cyclooxygenase enzyme (COX-1) [113]. Inhibition of COX-1 enzyme is associated with significant damage to the gastrointestinal tract, resulting in ulcers [120]. Because of these numerous effects of vitamin E, Bast and Haenen [113] called for a complete re-evaluation of the effect of vitamin E administration, suggesting that such evaluation should include studies of the metabolism and the biological effects of the metabolites of vitamin E.

*β*-Carotene, an important pro-vitamin which can be easily converted into retinol (vitamin A) by a dioxygenase enzyme has been shown to increase cancer incidence in both smokers and asbestos workers [121]. This is because *β*-carotene is only effective as an antioxidant at a low O_2_ tension, whereas at high O_2_ tensions it may even stimulate lipid peroxidation [122]. The unstable oxidised metabolites of *β*-carotene facilitate carcinogenesis by promoting DNA damage or by inducing cytochrome P450 enzymes that promote carcinogen activation through other xenobiotic metabolisms [123]. Gutteridge and Halliwell [1] postulated that free-radicals may not pose a great threat to our health unless we expose ourselves to an excess of free radical-generating agents such as cigarette smoke or ionising radiation.

In contrast to its antioxidant action, dihydrolipoic acid, a metabolic product of lipoic acid, can also function as a pro-oxidant. Similar to vitamins C and E, the pro-oxidant action of lipoic acid is mediated by the reduction of transition metals [113]. The biosynthesis of lipoic acid occurs from fatty acids and is present in meat and liver products. Under normal circumstances, lipoic acid operates as a cofactor by linking to the lysine residues of dehydrogenase multienzyme complexes [58]. It mediates the reactions by binding acyl groups and transferring them from one part of the enzyme complex to another thereby being reduced to dihydrolipoic acid which is reoxidized by lipoamide dehydrogenase with concomitant formation of NADH. Lipoic acid and its metabolite dihydrolipoic acid operate in this way as a redox couple, carrying electrons from the substrate of the dehydrogenase to NAD^+^ [113]. 

Caffeic acid, a widely used antioxidant, may also act as a pro-oxidant. Upon thermal treatment, caffeic acid was shown to produce decomposition products with significant pro-oxidant activity. In fact, highly reactive cations were generated during the early phases of caffeic acid degradation, affecting both the oxidative status and the reaction pathway of the system [70].

Knowledge on side effects, biotransformation or bio-kinetics of the natural products including antioxidants is helpful in preventing the administration of toxic products to unsuspecting consumers. However, large populations are already exposed to a number of supplemented foods, beverages and cosmetics before toxicity studies have been concluded. Gutteridge and Halliwell [1] have also highlighted that the public is becoming too familiar with the term ‘antioxidant’ and this has sown the seeds of belief in the minds of the general public that antioxidants might be the ‘elixirs’ of good wellbeing. Other negative effects of exogenous antioxidants include down-regulation of important endogenous antioxidants, interference with the immune system and a possible increase in microbial damage or the normal cellular protective responses to tissue damage. To prevent toxicity from antioxidants and other natural products, Bast and Haenen [113] put forward a few points to follow when directly involved in natural product research and processing. These include: (1) an urgent change in the approach used to evaluate food additives and supplements to include arbitrary safety factors which allows extrapolation of research results from rat to human, (2) understanding of the bio-kinetics, bioavailability and biotransformation of food and drug ingredients should be extended, (3) bio-kinetic/bio-efficacy modelling should be used to help in standardizing dosages, and (4) the risk/benefit analyses of all supplements that have health claims should be warranted. High-dose antioxidant supplements do no good and obviously cause harm to human health while low dose mixtures can sometimes be good but may be recommended only for undernourished populations [1].

## 7. Bioavailability of Antioxidant Molecules

The answer to the question on bioavailability is not easy to come by as there exists a body of contradicting data. From the small reservoir of data on bioavailability, it has been shown that the concentrations of intact flavonoids in human plasma rarely exceed 1 mM when 2.3 g phenolics per trial were ingested. These maximum concentrations are most often reached 1–2 h after ingestion except for polyphenols, which are absorbed only after partial degradation by the microflora in the colon. In contrast, phenolic compounds are believed to be responsible for the antioxidant capacity of some drinks. In spite of having a greater antioxidant capacity than ascorbic acid and α-tocopherol, some phenolic compounds have scanty bioavailability and therefore make less contribution to the antioxidant plasmatic capacity [124]. King [125] reported the bioavailability of flavonoids like catechin, quecetin and isoflavones to be about 10–50%. Vissers *et al*. [126] showed that absorption of virgin olive oil phenols is between 55–66% and of that amount, 5% is excreted in urine as tyrosol and hydroxytyrosol. 

What makes phenolic compounds difficult to absorb compared to other molecules? Phenolic compounds exist in many different chemical forms. Chemical structures greatly affect the reactivity and conjugation reactions of phenolic compounds with glucuronide, methyl and sulphate groups that are chiefly involved in absorption reactions. Most phenolic compounds usually exist as glycosylated forms. The types of sugars are usually glucose or rhamnose but can also be arabinose, galactose, xylose, glucuronic acid [29,79]. The glycosylation and the type of sugar conjugation greatly influence the biological properties of phenolics such as diffusion through membranes as gylcosylation lowers the membrane permeability of phenolic compounds [29,127]. It is therefore essential to remove the glucose moiety before a phenolic compound can easily be absorbed. The removal of the sugar moiety usually takes place in the food by the activation of glycosidase enzymes, in the gastrointestinal mucosa cells or the enzyme can be secreted by the colon microflora [29]. Other chemical reactions such as acylation and esterification reduce the membrane permeability of phenolic compounds. Other factors that affect bioavailability include the presence of fat in the diet, fiber content and heat treatments [6]. The presence of fat in the diet increases the bioavailability of lycopene. Many phenolic compounds are not bioavailable because of their low solubility, stability and may require biotransformation before they reach the systematic circulation [6].

Halliwell *et al*. [118] proposed that antioxidants such as flavonoids and other phenolic compounds could exert their antioxidant effects within the gastrointestinal tract without any need for absorption. This could account for the protective effects of phenolic compounds against gastric and colonic cancer. The authors cited the effects of green tea as an example. Green tea rapidly decreases prostaglandin E_2_ concentrations in the human rectal mucosa, leading to the inhibition of cyclooxygenase enzymes. Halliwell *et al*. [118] argued that the absorption of phenolic compounds is incomplete until they enter the colon where they exert their beneficial effects. The gastrointestinal tract is constantly exposed to reactive oxygen species such as chlorine and nitrogen species from the diet and/or products of bacterial fermentation. The stomach acts as a ‘bioreactor’ for such reactive oxygen species [118].

## 8. The Fate of the Oxidised Products (Mysteries in Antioxidant Reactions)

Several researchers have tried to reveal the mysteries surrounding antioxidant reactions. During the antioxidant reactions, electrons and hydrogen atoms are exchanged and at the end of the process, it is understood that another molecule has to be oxidised (*when an irresistible force meet with an immovable object, something has to give in*). The question that follows is; what is the fate of the oxidised molecules? It should be understood that the complexity and heterogeneity of biological systems does not allow anything to work in isolation, if a reaction takes place to oxidise one molecule then something must be reduced and there must be a balance of electrons [128]. Such complications have led to the dichotomy of antioxidants, mainly the fact that *in vitro* activity cannot easily be translated into *in vivo* effects [16,128]. 

A simple explanation which reveals the mysteries in antioxidant reactions has been put forward to explain the fate of oxidised products. The theory explains how two molecules work together by regenerating or “sparing” each other. Vitamins C and E work as antioxidants by quenching free radicals and providing hydrogen atoms (reducing equivalents). The donated hydrogen atoms then pair up with unpaired electrons on free radicals. In the process, the vitamins themselves become oxidized (spent). The active reduced forms of the vitamins are then regenerated (spared) by reduction with glutathione (GSH) and/or other reductants such as ascorbate, NADH/NADPH, dihydrolipoate (a thiol containing antioxidant like GSH), ubiquinol, or cytochrome transport electrons [74]. 

Ascorbic acid is believed to be regenerated from its oxidized form, dehydroascorbic acid, by reductase enzyme(s) that utilize GSH and probably NADPH as well (reducing equivalents) [74]. Another example is of lipoic acid, an antioxidant that operates as a cofactor to dehydrogenase multienzyme complexes [58]. Lipoic acid mediates the reactions by binding acyl groups and transferring them from one part of the enzyme complex to another thereby getting itself reduced to dihydrolipoic acid, which is reoxidized by lipoamide dehydrogenase to regenerate lipoic acid with concomitant formation of NADH [113].

From these studies, it can be deduced that several enzyme systems are involved in deciding the fate of the oxidised antioxidant molecules, leading to the regeneration of the original antioxidant molecules. Another deduction is that antioxidants cannot operate efficiently when isolated; instead they work in a synergistic manner, sparing each other during the reactions.

## 9. Are Antioxidants Mythical or Fascinating Biomolecules? Conclusions and Future Prospects

The existence of many research papers and reviews on the topic of antioxidants is a case in point. It provides support and evidence that antioxidants do exist and are not just mythical biomolecules but are responsible for a number of radical scavenging and chain-breaking reactions that keeps us healthy. An additional large body of literature outside the scientific publishing houses supports the role of antioxidants in the pathogenesis of human diseases. By acting as antioxidants, phenolic compounds, especially flavonoids, may improve cell survival; and as pro-oxidants, they induce apoptosis and prevent cancerous cell growth [9]. However, though antioxidants are fascinating and useful, they should be used with caution, at least until such time as enough research has been undertaken to ascertain their safety. Currently the majority of such papers simply concentrate on the presence of such compounds in plants used as food and medicinally.

Perhaps it would be helpful for researchers to concentrate on bioavailability, absorption, metabolism, pharmacokinetics and biotransformation studies of antioxidants. The extent to which the many interactions and synergisms demonstrated *in vitro* could actually occur *in vivo* remains to be determined and presents an important and formidable challenge to future researchers. Such studies could offer an answer to a question on whether an administered antioxidant could easily reach the site of increased free-radical formation.
